# Orexinergic pathway as a potential therapeutic candidate for the modulation of glucose homeostasis

**DOI:** 10.3389/fphys.2025.1659753

**Published:** 2025-12-08

**Authors:** Jean Claude Hakizimana, Pelagie Izabayo, Zephyrin Izukwizabigenza, Abdullateef Isiaka Alagbonsi

**Affiliations:** Department of Physiology, School of Medicine and Pharmacy, College of Medicine sand Health Sciences, University of Rwanda, Huye, Rwanda

**Keywords:** diabetes mellitus, glucose, hypothalamus, orexin, therapeutic target

## Abstract

**Background:**

Glucose homeostasis is regulated by both central and peripheral systems to maintain metabolic stability during periods of feeding, fasting, and stress. Orexins A and B, hypothalamic neuropeptides traditionally associated with arousal and feeding behavior, are increasingly recognized for their pivotal role in glucose homeostasis via neural and endocrine mechanisms. This review synthesizes the available data orexinergic-glucose pathway.

**Method:**

Following PRISMA guidelines, a systematic search of PubMed and Wiley Online Library identified original studies (between January 1999 and May 2025) examining orexin’s impact on glucose homeostasis in mammalian models. Molecular mechanisms were analyzed and grouped by tissues: central nervous system (CNS), pancreas, liver, skeletal muscle, adipose or vascular tissue, gut, and whole-body systems.

**Results:**

Thirty studies were included. Central orexin neurons integrate glycemic inputs via the autonomic nervous system (ANS). Orexin-A stimulates insulin secretion and β-cell proliferation through OX1R/PI3K/Akt/ERK1/2 signaling. It suppresses hepatic gluconeogenesis via PGC-1α downregulation and enhances insulin sensitivity. In adipose tissue, it promotes GLUT4 translocation and adiponectin via PPARγ/C/EBPα, while protecting endothelium from high-glucose damage via SIRT1/NLRP3 inhibition. In the gut, orexin inhibits SGLT-1-mediated glucose absorption through OX1R/OX2R. Systemic orexin deficiency induces insulin resistance, reversible by treatment.

**Conclusion:**

The orexinergic pathway serves as a metabolic integrator, linking central glucose sensing with peripheral utilization. Its context-dependent duality, promoting glucose release in hypoglycemia and insulin sensitivity in hyperglycemia, highlights a unique regulatory role. Orexin receptors are promising therapeutic targets for diabetes and metabolic syndrome. Sex-stratified human studies are needed, as preclinical data reveal marked sexual dimorphism in orexin-mediated glucose regulation, with males exhibiting greater metabolic vulnerability to orexin deficiency.

## 1 Introduction

The intricate orchestration of glucose homeostasis is paramount for maintaining cellular function and overall metabolic health, involving a complex interplay of hormonal, enzymatic, and neural pathways. While peripheral metabolic organs have historically been the primary focus of glucose homeostasis research, contemporary neuroscience has illuminated the crucial role of the central nervous system (CNS) in maintaining glucose balance (Choi and Kim, 2022). The brain acts as a conductor, orchestrating various peripheral organs involved in homeostatic processes to keep blood glucose levels within a normal range (Scheen, 2010). Within the brain, several nuclei are known to regulate glucose metabolism. However, more recent studies indicate that additional brain areas beyond these nuclei may also contribute to maintaining glucose homeostasis (Diepenbroek et al., 2013).

The hypothalamus, a critical brain region, regulates numerous physiological processes, including energy balance, feeding behavior, and glucose metabolism (Alonge et al., 2021). Neurons in this region have been found to govern glucose metabolism, even in the absence of insulin (Coppari, 2015). More specifically, neurons originating from the lateral hypothalamus (LH) have emerged as critical regulators of energy balance and glucose metabolism (Bae and Song, 2017) . Orexin neurons are localized primarily within the LH and project to many brain regions involved in arousal, feeding, and metabolism (Shiuchi et al., 2009; Yi et al., 2009; Otlivanchik et al., 2015).

Neural networks called orexinergic pathways or hypocretinergic pathways use the neuropeptides orexin A and B (also called hypocretin-1 and -2, respectively) to control several physiological processes. These hypothalamic neuropeptides, orexins A (hypocretin-1; 33 amino acids) and B (hypocretin-2; 28 residues), are produced by the prepro-orexin (pre-prohypocretin) precursor, encoded by the *HCRT* gene, and are implicated in the control of eating behavior, sleep-wake cycles, and neuroendocrine homeostasis (Messina, 2014). The precursor has 130 amino acids in rats and 131 residues in humans (Sakurai et al., 1998).

Orexins exert their effects through two G-protein-coupled receptors (GPCRs), orexin receptor 1 (OX1R, also known as HCRTR1) and orexin receptor 2 (OX2R, also known as HCRTR2), both belonging to the Class A (rhodopsin-like) family of GPCRs. OX1R displays higher affinity for orexin-A and couples primarily to Gq/11 proteins, activating phospholipase C (PLC) to increase intracellular Ca^2+^ mobilization and downstream pathways such as protein kinase C (PKC) and extracellular signal-regulated kinase (ERK). OX2R has equal affinity for both orexins and couples to Gq/11, Gi/o (inhibiting adenylyl cyclase and cAMP), and potentially Gs, enabling more versatile signaling, including β-arrestin-mediated ERK activation (Nowak et al., 2005). Tissue-specific expression is regulated by distinct promoters: for instance, specificity protein 1 (SP1) and NF-κB elements drive OX1R in pancreatic β-cells, while CREB-responsive sites predominate in hepatic OX2R expression. Orexin receptors are widely distributed, with high central expression in the hypothalamus and brainstem, and peripheral localization in tissues such as the pancreas (predominantly OX1R, 7-fold higher than OX2R), liver, skeletal muscle, adipose, and gut, where they modulate local metabolic responses (Nowak et al., 1999; Jöhren et al., 2001; Xu et al., 2011; Kukkonen, 2019). This indicates a potential role for orexin receptors in the regulation of glucose. Emerging evidence has shown that dysregulation in the orexinergic system impacts glucose homeostasis, a critical aspect of metabolic health (Barson and Leibowitz, 2017).

Among the integration and regulation of homeostatic systems, control of energy balance is one of the most intricate hypothalamic duties. Other brain regions and chemicals released by peripheral organs, such as the gastrointestinal tract and the adrenal glands, must work in concert to accomplish this. The peripheral signals inform the CNS about the condition of the energy stores, which influences energy intake and expenditure (Yamanaka et al., 2006). It is also well-established that metabolic disorders, including diabetes mellitus (DM) and obesity, involve disruption of the intricate balance between energy intake and expenditure (Inoki et al.,2012). Despite various advances in research on glucose homeostasis, DM is still a global burden. The latest atlas of the International Diabetes Federation (IDF) shows that 588.7 million people, corresponding to 11.1% (or 1 in 9) of the adult population (between 20 and 79 years), were living with DM in 2024, with over 4 in 10 unaware that they have the condition. The IDF also projected that 853 million people (corresponding to 1 in 8 adults) will be living with DM by 2050, representing a 46% increase worldwide (IDF, 2025). Yet, there is presently no cure for DM, as most of the available treatments act via peripheral regulation of glucose homeostasis, which mitigates symptoms to improve the quality of life of the patients. Thus, current scientific efforts have been exploring the role of mediators that act both centrally and peripherally to regulate glucose homeostasis, creating an avenue to identify agents that can stabilize glucose levels via insulin-dependent and insulin-independent mechanisms. Interestingly, the orexinergic pathway is a potential candidate that fits into this criterion, and it may help to improve diagnostic strategies and develop targeted therapeutic approaches for individuals with DM.

A substantial body of research has elucidated the role of orexin in regulating arousal, feeding, and energy homeostasis, with early studies establishing its central actions in the hypothalamus (Sakurai et al., 1998). Subsequent investigations expanded on these findings, demonstrating orexin’s involvement in peripheral metabolic processes, including glucose regulation (Tsuneki et al., 2010; Rani et al., 2018). While prior reviews have provided broad overviews of orexin’s functions in energy balance and motivated behaviors (Sakurai, 2005; Messina, 2014), they often lack tissue-specific mechanistic details in the context of glucose homeostasis. This systematic review builds upon these foundations by synthesizing experimental evidence on orexin’s central and peripheral roles in glucose homeostasis, offering a comprehensive, tissue-organized analysis to address gaps in understanding its therapeutic potential for metabolic disorders.

## 2 Methodology

This systematic review employed a narrative synthesis within a rigorous systematic framework, adhering to the Preferred Reporting Items for Systematic Reviews and Meta-Analyses (PRISMA) guidelines (Page et al., 2021) and incorporating elements of the PRISMA Extension for Scoping Reviews (PRISMA-ScR) (Tricco et al., 2018), where appropriate, to accommodate the broad, exploratory nature of mapping orexin’s role across multiple physiological systems. This dual approach enabled a comprehensive integration of evidence spanning molecular, cellular, and whole-organism levels, with particular emphasis on experimental studies that directly link orexinergic signaling to mechanisms of glucose homeostasis. By combining systematic rigor with narrative depth, the review aimed to not only quantify the volume and quality of evidence but also to elucidate mechanistic pathways, tissue-specific effects, and translational implications in a coherent, biologically meaningful narrative.

### 2.1 Search strategy

A comprehensive literature search was conducted across two major academic databases, PubMed and Wiley Online Library, covering the period from January 1999, the year following the discovery of orexins (Sakurai et al., 1998), through May 2025, to capture the full evolution of research in this field. The search strategy was designed to maximize sensitivity while maintaining specificity, using a structured Boolean combination of terms related to orexin signaling and glucose metabolism. Core orexin-related terms included “hypocretin,” “orexin-A,” “orexin-B,” “OX1R,” “OX2R,” “HCRTR1,” “HCRTR2,” and “orexin receptor,” while glucose homeostasis terms encompassed “glucose homeostasis,” “glucose regulation,” “insulin secretion,” “glucose uptake,” “gluconeogenesis,” “glycogenolysis,” “GLUT4,” “insulin sensitivity,” “hepatic glucose production,” “beta-cell function,” “counter-regulatory response,” “hypoglycemia,” and “hyperglycemia.”

The search was deliberately expanded to include tissue-specific keywords that reflected both central and peripheral sites of orexin action. For central mechanisms, terms such as “hypothalamus,” “lateral hypothalamus,” “LH orexin,” “perifornical area,” “central orexin,” “autonomic nervous system,” “sympathetic nervous system,” “SNS,” “parasympathetic,” “PNS,” “vagus nerve,” and “nucleus tractus solitarius” were incorporated. For peripheral tissues, keywords included “pancreas,” “pancreatic islet,” “beta cell,” “INS-1,” “hepatocyte,” “liver,” “skeletal muscle,” “muscle glucose uptake,” “adipose tissue,” “adipocyte,” “white adipose,” “brown adipose,” “gut,” “intestine,” “duodenum,” “jejunum,” “enterocyte,” “gut-brain axis,” “vascular endothelium,” and “endothelial cell.”

Further refinement ensured mechanistic and contextual depth through inclusion of signaling and regulatory terms: “PI3K,” “Akt,” “ERK1/2,” “AMPK,” “mTOR,” “GLUT4 translocation,” “insulin resistance,” “HOMA-IR,” “glucose tolerance test,” “hyperinsulinemic-euglycemic clamp,” “circadian rhythm,” “clock gene,” “sexual dimorphism,” “sex difference,” “male,” “female,” “estrogen,” and “androgen.” No language restrictions were applied during the initial electronic search to avoid missing potentially relevant non-English studies; however, only full-text articles available in English were included in the final analysis to ensure accurate interpretation and data extraction.

### 2.2 Inclusion and exclusion criteria

Study inclusion was governed by a stringent set of predefined criteria to ensure relevance, scientific rigor, and mechanistic insight. Eligible studies were required to be original experimental research published between January 1999 and May 2025, conducted in mammalian models (including rodents, humans, non-human primates, or derived cell lines), and to report direct intervention or observation involving orexin-A, orexin-B, OX1R, OX2R, or genetic/pharmacological manipulation of the orexin system (e.g., knockout, knockdown, receptor antagonists, or agonists). The primary outcome of interest was a quantifiable or mechanistically described effect on glucose homeostasis, including but not limited to changes in blood glucose concentration, insulin secretion or sensitivity, hepatic glucose production, muscle or adipose glucose uptake, β-cell proliferation or survival, or counter-regulatory hormonal responses. Studies were required to provide sufficient methodological detail and quantitative or qualitative mechanistic data to allow pathway mapping.

Studies were excluded if they were review articles, editorials, hypotheses, or commentaries; if they focused on non-orexin neuropeptides (e.g., NPY, AgRP) without orexin-specific data; if glucose homeostasis was not a measured outcome (e.g., feeding behavior studies without glycemic endpoints); or if they lacked mechanistic insight (e.g., purely correlative clinical observations). Non-mammalian models and studies with incomplete methodological reporting were also excluded to maintain biological relevance and reproducibility. (Table 1).

**TABLE 1:** Inclusion and exclusion criteria for articles.

Criteria	Included	Excluded
Publication date	January 1999 to May 2025	Before 1999
Language	English	Non-English
Study type	Original experimental studies	Reviews, meta-analyses, abstracts, and editorials
Intervention	Involves Orexin A/B, OX1R, or OX2R	Non-orexin-related studies
Outcomes measures	Glucose homeostasis	Non-glucose-related outcomes
Model Type	Humans, rodents, and cell lines	Non-mammalian models
Focus	Direct link with orexin and glucose homeostasis	Studies of orexin without metabolic context
Data quality	Randomized controlled studies and appropriate control groups	No control groups
Glucose regulation scope	Central and/or peripheral regulation involving known mechanisms	Studies that do not investigate the mechanism of action or metabolic impact

### 2.3 Study selection and data extraction

The study selection process was executed with systematic rigor, transparency, and reproducibility by two independent reviewers, with a third reviewer available to resolve any discrepancies through consensus. Following automated deduplication of records retrieved from PubMed and Wiley Online Library, all unique citations underwent initial title and abstract screening against the predefined eligibility criteria. Studies deemed potentially relevant progressed to full-text retrieval and in-depth assessment, during which reasons for exclusion were meticulously documented and categorized, such as absence of a glucose homeostasis outcome, non-experimental design, or insufficient mechanistic detail. This two-stage screening approach ensured comprehensive capture of pertinent evidence while minimizing inclusion of irrelevant or low-quality studies.

Data extraction was conducted using a standardized, piloted electronic form to guarantee consistency, completeness, and comparability across studies. Extracted fields encompassed study identification (first author, publication year, journal, DOI), study design and model characteristics (species, strain, sex, age, metabolic state such as fasting or diabetic, and sample size), intervention specifics (orexin isoform A or B, dose, route of administration whether intracerebroventricular, intraperitoneal, or *in vitro* receptor specificity, and use of antagonists, agonists, or genetic models), primary glucose-related outcomes (quantitative endpoints including fasting glucose, area under the curve in glucose tolerance tests, insulin levels, HOMA-IR, or clamp-derived glucose infusion rates), mechanistic data (intracellular signaling pathways such as phosphoinositide-3 kinase (PI3K)/protein kinase B (Akt), extracellular signal-regulated kinase (ERK)1/2, adenosine monophosphate-activated protein kinase (AMPK), or mechanistic target of rapamycin (mTOR); autonomic nervous system involvement; transcription factors; gene expression changes; and glucose sensing mechanisms), sex-specific findings (differences in orexin effects or receptor expression between males and females), and quality indicators (randomization, blinding, statistical methods, and reported limitations). This structured extraction framework not only facilitated direct cross-study comparisons but also enabled the construction of detailed, integrative mechanistic maps, visually synthesizing signaling cascades, transporter dynamics, and transcriptional regulation across tissues (Supplementary Material S1).

### 2.4 Quality and risk of bias assessment

Methodological quality and risk of bias were rigorously evaluated using a dual-assessment strategy tailored to study type, ensuring a nuanced appraisal of evidence reliability. For animal studies, the SYRCLE’s Risk of Bias (RoB) tool was applied, systematically examining domains including sequence generation, baseline characteristics, allocation concealment, random housing, blinding of investigators and outcome assessors, random outcome assessment, incomplete outcome data, and selective reporting (Hooijmans et al., 2014). For human clinical or interventional studies, the Cochrane Risk of Bias 2 (RoB 2) tool was employed, assessing bias arising from the randomization process, deviations from intended interventions, missing outcome data, measurement of the outcome, and selection of the reported result (Minozzi et al., 2022).

Each study received an overall classification of low, moderate, or high risk of bias, supported by domain-specific judgments that informed a narrative summary of methodological strengths and limitations. Notably, no studies were excluded solely based on quality; instead, risk of bias findings were transparently integrated into result interpretation and explicitly addressed in the limitations section. This inclusive yet critical approach provided a balanced, evidence-based assessment of the literature’s robustness, enhancing the review’s credibility and utility for translational inference (Supplementary Material S2).

### 2.5 Data synthesis and analysis

Given the substantial heterogeneity in study designs, experimental models, interventions, and outcome measures, a narrative synthesis was selected over meta-analysis to enable rich, thematic, and mechanistic integration of the evidence. Studies were organized thematically by the primary tissue system of orexin action: central neural regulation, pancreatic β-cell function, hepatic glucose metabolism, skeletal muscle glucose uptake, adipose and vascular tissues, the gut-orexin-glucose axis, and multi-systemic or supportive studies, with findings within each subsection synthesized both chronologically and mechanistically to trace the evolution of scientific understanding and highlight convergent signaling pathways.

Particular emphasis was placed on cross-cutting biological themes critical to orexin’s role in glucose homeostasis. These included glucose sensing in orexin neurons, distinguishing direct metabolic mechanisms for instance ATP-dependent KATP channel modulation from indirect pathways including vagal or hormonal inputs, AMP-dependent AMPK activation, or metabolite-surrogate sensing via lactate, alongside neuronal heterogeneity (glucose-inhibited versus glucose-excited subpopulations); receptor-level signaling, delineating differential roles of OX1R (primarily Gq/PLC/Ca^2+^-mediated) versus OX2R (Gq/Gi/Gs and β-arrestin pathways) and their tissue-specific coupling; transcriptional regulation, identifying key transcription factors modulated by orexin pathways (e.g., CREB, FOXO1, PPARγ, PDX1, PGC-1α, SREBP-1c) and their downstream effects on metabolic gene expression; and contextual modulators, including circadian rhythmicity, sexual dimorphism, metabolic state (fasting, diabetes, obesity), and stress.

Mechanistic pathways were visually consolidated, with comprehensive legends detailing receptor activation, second messenger systems, kinase cascades, transporter trafficking (GLUT4), and transcriptional outputs. Sexual dimorphism was highlighted wherever data permitted, noting differential receptor expression, signaling sensitivity, or metabolic outcomes between males and females, while circadian influences were woven throughout to explain time-of-day variability in orexin effects and inform therapeutic timing, such as suvorexant administration. This structured, multilevel synthesis directly establishes a robust foundation for identifying knowledge gaps, generating therapeutic hypotheses, and guiding future experimental and clinical investigations into orexin as a dynamic modulator of glucose homeostasis.

## 3 Results

**FIGURE 1:**
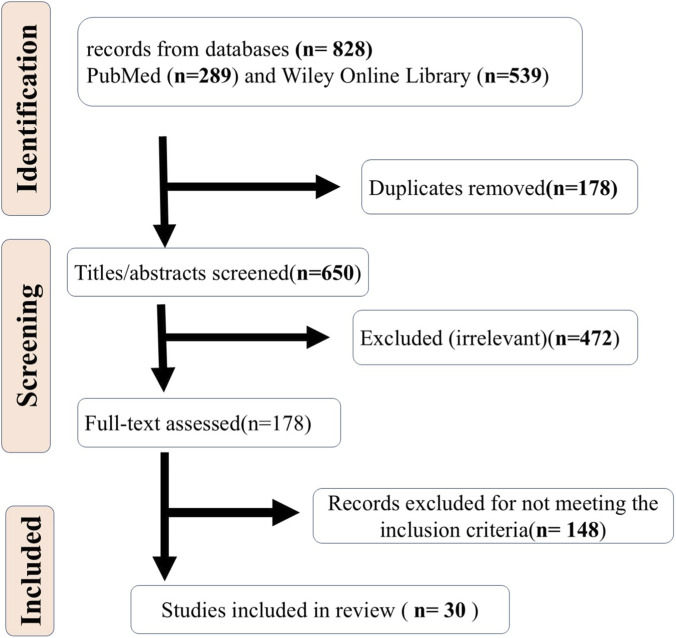
PRISMA flow diagram for article selection.

Initially, 828 records were retrieved (289 from PubMed, 539 from Wiley Online Library). After removing 178 duplicates, 650 remained for title/abstract screening. Of these, 472 were excluded as irrelevant, leaving 178 for full-text assessment. Ultimately, 148 records were excluded for not meeting the inclusion criteria, and 30 studies were included in the review (Figure 1).

The 30 included studies spanned a diverse range of experimental models, comprising animal studies (n = 23), cellular investigations (n = 6), and one human clinical trial (n = 1). Collectively, these investigations examined the effects of orexin-A, orexin-B, or their receptors (OX1R and OX2R) on direct indices of glucose homeostasis, such as blood glucose levels, insulin secretion, hepatic glucose production, and GLUT4 translocation, as well as indirect indices, including glucose sensing within orexin neurons and hypoglycemia-triggered arousal or feeding behavior. To facilitate mechanistic interpretation, the studies were systematically categorized into six tissue- or organ-system-based groups, as summarized in Table 2.

**TABLE 2:** Mechanistic classification of the 30 included studies.

Category	N	Key focus	Representative studies	Core mechanisms	Transcription factors (TFs)
Central neural regulation	15	Glucose sensing; hypoglycemia activation; ANS-mediated peripheral control; feeding behavior	Griffond et al. (1999), Moriguchi et al. (1999), Cai et al. (2001), Liu et al. (2001), Burdakov et al. (2006), Tkacs et al. (2007), González et al. (2008), Tsuneki et al. (2008), Shiuchi et al. (2009), Gonzàlez et al. (2009), Yi et al. (2009), Yang et al. (2018), Teegala et al. (2020), Teegala et al. (2023), Devère et al. (2025)	Non-metabolic GI sensing via K_2_P/SGLT; Fos/mRNA upregulation in hypoglycemia; OX1R → sympathetic increase in EGP; Gαi/o-cAMP-PKA → VTA glutamate/dopamine; AMPK/T-type Ca^2+^ channel in restriction	*c-Fos* (hypoglycemia); CREB (PKA)
Pancreatic β-Cell function	6	Insulin secretion; β-cell proliferation/survival	Ouedraogo et al. (2003), Nowak et al. (2005), Chen et al. (2013), Park et al. (2015), Kaczmarek et al. (2017), Skrzypski et al. (2016)	OX1R → PI3K/Akt/ERK (proliferation); TRP/Ca^2+^ channel and cAMP/EPAC2/RyR (GSIS); glucose downregulation of orexin release	FOXO1↓, Elk-1, CREB, c-Fos
Hepatic glucose metabolism	3	Gluconeogenesis downregulation; glucose uptake/oxidation	Tsuneki et al. (2015), Tsuneki et al. (2016), Liu et al. (2016)	PI3K/Akt/mTOR → GLUT1/PDH; ANS → PEPCK/PGC-1α downregulation; circadian orexin antagonism	FOXO1↓, PGC-1α↓
Adipose and vascular tissue	2	Glucose uptake; endothelial protection	Skrzypski et al. (2011), Zhang et al. (2019)	PI3K → GLUT4/PPARγ; SIRT1 → ROS/NLRP3 downregulation	C/EBPα, NF-κB↓, FOXO1↓
Gut–Orexin–Glucose axis	1	Intestinal absorption downregulation	Ducroc et al. (2007)	OX1R (epithelial), OX2R (neuronal/CCK) → SGLT-1 downregulation	—
Multi-System/Whole-Body	3	Systemic tolerance; insulin resistance	Tsuneki et al. (2002), Zarifkar et al. (2017), Devère et al. (2025)	Akt/GSK3β; neural/hormonal utilization	GSK3β↓

Akt, protein kinase B; ANS, autonomic nervous system; AMPK, adenosine monophosphate (AMP)-activated protein kinase; C/EBPα, cytosine-cytosine-adenosine-adenosine-thymidine (CCAAT)/enhancer binding protein alpha; cAMP, cyclic adenosine monophosphate; CCK, cholecystokinin; CREB, cAMP, response element-binding protein; EGP, endogenous glucose production; EPAC2, exchange protein directly activated by cAMP, 2; ERK, extracellular signal-regulated kinase, FOXO1, Forkhead Box O1, GI, glucose-inhibited; GSIS, glucose-stimulated insulin secretion; GSK3β, glycogen synthase kinase-3, beta; GLUT, glucose transporter; K_2_P, tandem-pore potassium channel; mRNA, messenger ribonucleic acid; mTOR, mechanistic target of rapamycin; NF-κB, nuclear factor kappa B; NLRP3, NOD-, LRR-, and pyrin domain-containing protein 3; OX1R/OX2R, orexin receptor 1/2; PDH, pyruvate dehydrogenase; PEPCK, phosphoenolpyruvate carboxykinase; PGC-1α, peroxisome proliferator-activated receptor gamma coactivator 1-alpha; PI3K, phosphoinositide 3-kinase; PKA, protein kinase A; PPARγ, peroxisome proliferator-activated receptor gamma; ROS, reactive oxygen species; RyR, ryanodine receptor; SGLT, sodium-glucose cotransporter; SIRT1, sirtuin 1; TFs, transcription factors; TRP, transient receptor potential; VTA, ventral tegmental area.

### 3.1 Central regulation of glucose by orexin

**FIGURE 2:**
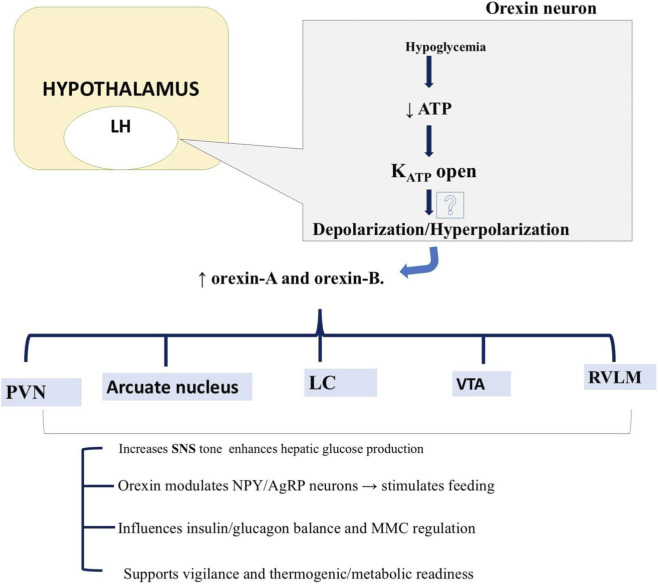
Hypoglycemia activates orexin neurons in the lateral hypothalamus (LH). The orexin neurons are distributed to different brain nuclei like the arcuate nucleus, paraventricular nucleus (PVN), ventral tegmental area (VTA), locus coeruleus (LC), and rostral ventrolateral medulla (RVLM) for maintaining glucose levels. SNS, sympathetic nervous system; NPY/AgRP, Neuropeptide Y/Agouti-related peptide; MMC, migrating motor complex.

Orexin neurons in the lateral hypothalamus (LH) serve as critical integrators of glycemic and metabolic signals, orchestrating counter-regulatory responses through direct glucose sensing, afferent neural inputs, and efferent autonomic and behavioral pathways. The 15 studies in this category collectively revealed a context-dependent duality in orexin function: robust activation during hypoglycemia to mobilize glucose, enhance arousal, and drive feeding behaviors, contrasted with suppression or pharmacological antagonism in hyperglycemic or diabetic states to improve insulin sensitivity and glucose utilization (Figure 2).

#### 3.1.1 Afferents and glucose access to LH-Orexin neurons

LH-orexin neurons are strategically positioned to receive convergent inputs from multiple energy-sensing brain regions, enabling rapid integration of peripheral metabolic status. The arcuate nucleus (ARC) provides orexigenic drive via NPY/AgRP neurons and anorexigenic modulation through pro-opiomelanocortin (POMC) neurons, directly influencing orexin excitability. The nucleus of the solitary tract (NTS) and dorsal motor nucleus of the vagus (DMV) relay visceral and glycemic signals from the periphery, as demonstrated by Cai et al. (2001), who showed that NTS-mediated vagal afferents are essential for hypoglycemia-induced orexin activation. Serotonergic projections from the raphe nuclei fine-tune orexin responses to peripheral glucose fluctuations via OX1R and OX2R signaling, while catecholaminergic inputs from the brainstem exert complex excitatory and inhibitory effects, often mediated by noradrenaline (Yamanaka et al., 2006).

#### 3.1.2 Intrinsic glucose sensing: predominantly glucose-inhibited (GI)

Glucose reaches LH-orexin neurons primarily through direct diffusion across the blood-brain barrier near the third ventricle, where physiological extracellular concentrations range from 1 to 5 mM. This direct access supports rapid, real-time sensing. Orexin neurons are overwhelmingly glucose-inhibited (GI), meaning they hyperpolarize and reduce firing as extracellular glucose rises, thereby suppressing arousal and feeding during energy surplus. This GI dominance ensures predictive homeostasis, allowing behavioral adaptation before full metabolic processing. Indirect pathways also contribute: tanycytes lining the ventricle may shuttle glucose or its metabolites, and NTS relays transmit systemic glycemic changes. Overall, sensing is predominantly extracellular and non-metabolic, bypassing intracellular ATP generation and relying instead on membrane-level detection mechanisms (Burdakov et al., 2006).

Burdakov et al. (2006) established that tandem-pore K^+^ (K_2_P) channels are the primary mediators of this inhibition: physiological glucose levels (1–5 mM) trigger K^+^ efflux via extracellular binding, independent of ATP, calcium, or intermediary metabolism. González et al. (2008) confirmed the non-metabolic nature of this sensing by showing that non-metabolizable glucose analogs such as 2-deoxyglucose (2-DG) and mannose fully mimic native glucose in inducing hyperpolarization through K^+^ currents, while glucokinase inhibitors have no effect. In a follow-up, González et al. (2009b) demonstrated that GI sensing in orexin neurons operates via signaling-independent pathways, with 2-DG replicating glucose effects and ruling out ATP-sensitive potassium (KATP) channel involvement, unlike in pancreatic β-cells. Glucose-excited (GE) responses, mediated by electrogenic Na^+^-glucose cotransport (SGLT), were observed in adjacent hypothalamic neurons but not in orexin cells themselves.

Further resilience in the system was revealed by González et al. (2009a), who found that genetic deletion of TWIK-related acid-sensitive K^+^ channels (TASK) 1 and 3 disrupted baseline excitability and firing rates but left glucose sensing fully intact, indicating functional redundancy among K^+^ leak channels. Complementing this, Liu et al. (2001) showed that orexin-A itself potently excites glucose-sensitive LH neurons, increasing firing rates by up to 500% in a postsynaptic, tetrodotoxin-resistant manner, suggesting an autoregulatory loop that amplifies low-glucose detection.

#### 3.1.3 Hypoglycemia-induced activation

Acute hypoglycemia triggers a rapid and robust activation of the orexin system as part of the counter-regulatory response. Cai et al. (2001) provided seminal evidence: insulin-induced hypoglycemia, causing a >50% drop in plasma glucose, led to a 10-fold increase in hypothalamic orexin-B peptide levels and Fos expression in approximately 14% of orexin neurons. This activation was mediated by NTS relays of vagal afferents and was inhibited by feeding, particularly gastric distension, highlighting orexin’s role in hunger-driven restoration. Earlier foundational studies laid the molecular groundwork: Moriguchi et al. (1999) reported a 33% increase in Fos-like immunoreactivity in LH orexin neurons during hypoglycemia, while Griffond et al. (1999) observed upregulation of prepro-orexin mRNA, indicating transcriptional activation within hours.

Extending these molecular changes to functional outcomes, Tkacs et al. (2007) demonstrated that hypoglycemia at 2.4–2.6 mM doubled wake time to 48%, fragmented non-REM sleep, and increased Fos expression in orexin neurons from 8.7% to 37% directly linking orexin activation to heightened arousal and vigilance during energy crises.

#### 3.1.4 Behavioral and autonomic efferents

Under conditions of energy restriction or pathological hyperglycemia, orexin neurons drive motivated behaviors and peripheral glucose mobilization via well-defined efferent pathways.

In obese models, Yang et al. (2018) reported that orexin-A modulates glucose-responsive arcuate neurons through OX1R and CB1R co-signaling, increasing food intake and altering neuronal firing patterns. Teegala et al. (2020) found that low glucose (0.25–0.7 mM) stimulates orexin GI neurons to increase ventral tegmental area (VTA) glutamatergic transmission, elevating the AMPA/NMDA receptor ratio and reinforcing reward-based feeding. In a complementary study, Teegala et al. (2023) further showed that calorie restriction activates orexin neurons through AMPK-dependent depolarization and T-type Ca^2+^ channel opening, projecting to the VTA to enhance dopamine release and food-seeking behavior; ghrelin further amplifies this via PKA signaling.

On the autonomic front, Yi et al. (2009) demonstrated that perifornical orexin disinhibition activates sympathetic outflow via OX1R, upregulating hepatic phosphoenolpyruvate carboxykinase (PEPCK) and glucose-6-phosphatase to increase endogenous glucose production (EGP) and induce insulin resistance. Chronic orexin deficiency, as modeled by Tsuneki et al. (2008), leads to age-related insulin resistance through disrupted hypothalamic and peripheral Akt/GSK3β signaling, impairing glucose tolerance. Most recently, Devère et al. (2025) revealed that orexin knockout mice exhibit impaired glucose tolerance, reduced insulin sensitivity, and dysregulated hepatic gluconeogenesis, with sex-dimorphic effects, more severe obesity, and metabolic disruption in males, suggesting sexually divergent roles in central-peripheral coupling.

In summary, orexin neurons function as GI sensors that detect rising glycemia through non-metabolic, K_2_P channel-mediated mechanisms, suppressing activity during energy abundance. In hypoglycemia, NTS afferents trigger rapid Fos and c-Fos upregulation, activating orexin to drive arousal, motivated feeding, and sympathetic EGP via VTA dopamine/glutamate and hepatic OX1R pathways. Chronic orexin deficiency disrupts Akt/GSK3β signaling, leading to systemic insulin resistance. This dual regulatory profile mobilization in deficit and sensitivity in excess positions orexin as a high-value therapeutic target: OX1R/OX2R agonists could counter hypoglycemia unawareness in diabetes, while dual orexin receptor antagonists (DORAs) like suvorexant may improve insulin sensitivity in type 2 diabetes, particularly when timed to circadian rhythms.

### 3.2 Pancreatic regulation by orexin

Six studies collectively illustrated orexin’s insulinotropic and β-cell protective roles, mediated predominantly through the OX1R and exhibiting glucose-dependent modulation that aligns with physiological needs, enhancing insulin output during nutrient abundance while favoring glucagon release in fasting states. This dual capacity positions orexin as a fine-tuned regulator of pancreatic endocrine function, with potential therapeutic parallels to incretin mimetics.

Orexin-A directly stimulates β-cell proliferation and survival through canonical survival pathways. In INS-1E rat insulinoma cells, Skrzypski et al. (2016) showed that orexin-A at 100 nM activates extracellular signal-regulated kinase 1/2 (ERK1/2) to drive cell cycle progression and proliferation, while independently engaging transient receptor potential (TRP) channels to elevate intracellular calcium and trigger insulin secretion effects that were unaffected by changes in insulin mRNA, indicating post-transcriptional regulation. Complementing this, Chen et al. (2013) demonstrated in the same cell line that orexin-A acts via OX1R-coupled PI3K/Akt signaling to enhance cell viability, boost insulin content, and suppress apoptosis, with downstream inhibition of the pro-apoptotic transcription factor, Forkhead Box O1 (FOXO1), playing a central role in β-cell preservation.

At the functional level, orexin-A potentiates glucose-stimulated insulin secretion (GSIS) through second-messenger amplification. Park et al. (2015) observed in perifused mouse islets and intact animals that subcutaneous orexin-A enhances GSIS in a cAMP-dependent manner, involving EPAC2 activation of ryanodine receptors (RyR) to mobilize intracellular calcium stores. This resulted in a rapid insulin surge followed by delayed leptin elevation from adipose tissue, suggesting coordinated postprandial metabolic signaling. In a translational context, Kaczmarek et al. (2017) administered chronic orexin-A to streptozotocin/high-fat diet-induced type 2 diabetic rats and reported improved glucose control and β-cell survival, mediated by reductions in circulating tumor necrosis factor-alpha (TNF-α) and non-esterified fatty acids (NEFA), highlighting orexin’s anti-inflammatory and anti-lipotoxic effects in diabetic stress.

Receptor pharmacology further clarifies orexin’s insulinotropic hierarchy. Nowak et al. (2005) perfused isolated rat pancreatic islets and found that both orexin-A and orexin-B stimulate insulin release in a concentration-dependent manner at both 6.66 mM and 26.4 mM glucose, with orexin-A being markedly more potent due to higher affinity for OX1R, which dominates pancreatic expression (sevenfold over OX2R); both receptors converge on phospholipase C (PLC)/calcium signaling. Finally, Ouedraogo et al. (2003) revealed a feedback loop within the endocrine pancreas: low glucose (2.8 mM) triggers calcium-dependent release of orexin-A from islet cells, which in turn promotes glucagon secretion while suppressing GSIS, thereby prioritizing counter-regulatory hormone output during fasting, a mechanism that mirrors orexin’s central role in hypoglycemia.

**FIGURE 3:**
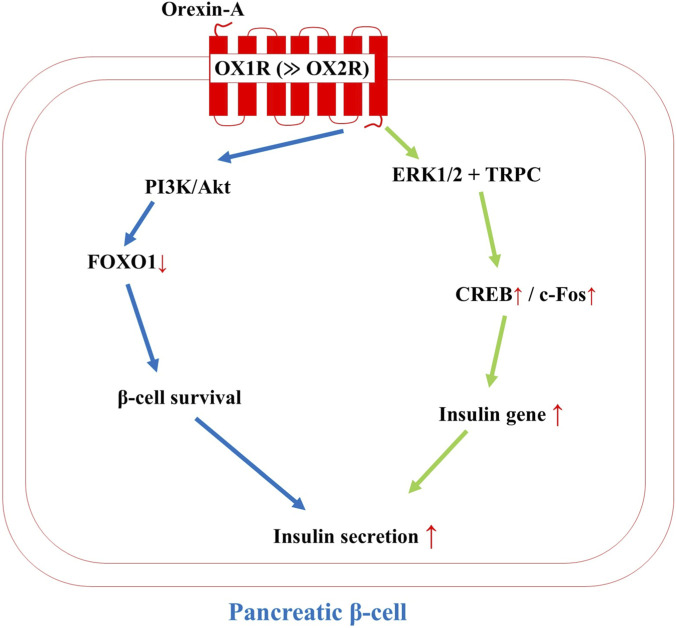
Orexin-A acts predominantly through orexin receptor 1 (OX1R ≫ OX2R) to stimulate both acute insulin secretion and long-term β-cell survival and proliferation. Key downstream pathways include PI3K/Akt-mediated FOXO1 inactivation and ERK1/2–CREB signaling. OX1R, orexin receptor 1; PI3K, phosphoinositide 3-kinase; Akt (PKB), protein kinase B; FOXO1, forkhead box protein O1; ERK1/2, extracellular signal-regulated kinases 1/2; CREB, cAMP-response element-binding protein; TRPC, transient receptor potential canonical channel.

In summary, orexin enhances β-cell mass and survival via PI3K/Akt/ERK signaling and FOXO1 suppression, while amplifying GSIS through cAMP/EPAC2/RyR and TRP/calcium pathways, often involving CREB-mediated transcriptional support. However, under low-glucose conditions, pancreatic orexin-A release promotes glucagon at the expense of insulin, ensuring glycemic defense. OX1R-selective agonists could thus mimic GLP-1–like effects, boosting β-cell function and resilience in type 2 diabetes while avoiding the central arousal side effects of non-selective compounds (Figure 3).

### 3.3 Hepatic glucose metabolism

Three studies delineate orexin’s bidirectional influence on hepatic glucose handling, establishing its role in orchestrating daily glycemic rhythms through autonomic coordination and in suppressing pathological gluconeogenesis when antagonized, particularly under circadian-aligned timing in diabetic models.

Central orexin exerts tonic control over hepatic glucose production (EGP) via the autonomic nervous system (ANS). Tsuneki et al. (2015) demonstrated in wild-type and orexin-deficient mice that hypothalamic orexin-A generates daily oscillations in EGP by dynamically balancing sympathetic activation (which upregulates gluconeogenesis) and parasympathetic restraint (which enhances insulin sensitivity and suppresses Pepck expression). This bidirectional ANS modulation prevents hepatic insulin resistance and reduces endoplasmic reticulum stress, revealing orexin as a central conductor of metabolic circadian rhythmicity.

At the cellular level, orexin-A directly reprograms hepatocyte glucose flux toward oxidation and away from futile cycling. In human Hep3B hepatocellular carcinoma cells, Liu et al. (2016) showed that orexin-A activates PI3K/Akt/mTOR signaling to increase GLUT1 expression and glucose uptake, while simultaneously enhancing pyruvate dehydrogenase (PDH) activity, shifting metabolism from glycolysis to oxidative phosphorylation. These effects involve transcriptional repression of FOXO1 and PGC-1α, key drivers of gluconeogenic gene expression, with the PDH activation occurring independently of mTOR, indicating parallel signaling branches.

Translating antagonism to therapy, Tsuneki et al. (2016) administered the dual orexin receptor antagonist suvorexant during the rest phase in db/db type 2 diabetic mice and observed marked improvement in glucose tolerance, driven by downregulation of hepatic PEPCK and PGC-1α, reduced triglyceride accumulation, and lowered TNF-α expression. This circadian-specific suppression of gluconeogenesis aligned with enhanced sleep quality, suggesting that timed orexin blockade can simultaneously address metabolic and sleep dysfunction in diabetes.

**FIGURE 4:**
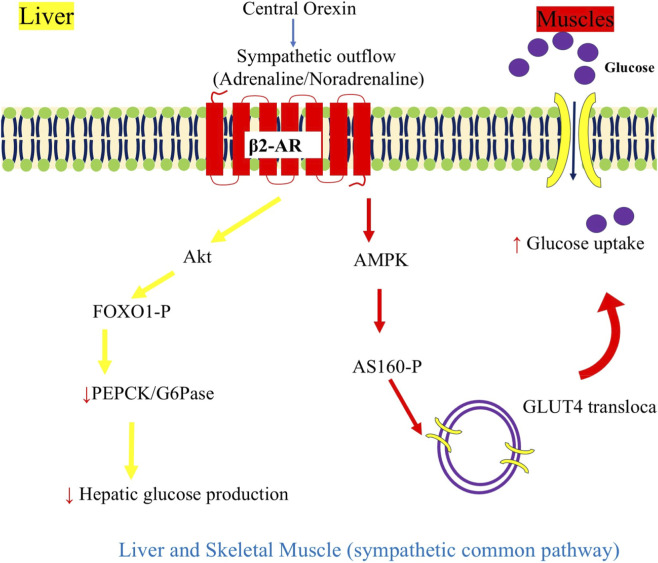
Central orexin release drives sympathetic outflow that simultaneously suppresses hepatic glucose production and enhances skeletal muscle glucose uptake. β2-AR, β2-adrenergic receptor; PKA, protein kinase A; Akt, protein kinase B; FOXO1, forkhead box protein O1; PEPCK, phosphoenolpyruvate carboxykinase; G6Pase, glucose-6-phosphatase; AMPK, AMP-activated protein kinase; AS160 (TBC1D4), Akt substrate of 160 kDa; GLUT4, glucose transporter type 4.

In summary, orexin sustains physiological hepatic glucose rhythmicity through ANS-mediated bidirectional control and PI3K/Akt/mTOR-driven substrate partitioning, with FOXO1 and PGC-1α as key transcriptional nodes (Figure 4). In diabetic hyperglycemia, rest-phase orexin antagonism (e.g., suvorexant) suppresses pathological gluconeogenesis and inflammation, offering a dual-benefit strategy improving glycemia while preserving sleep, ideal for nocturnal hepatic overactivity in type 2 diabetes.

### 3.4 Adipose and vascular tissue effects

Two studies highlight orexin’s insulin-mimetic and vasculoprotective actions in peripheral metabolic tissues, revealing its capacity to enhance glucose disposal in adipocytes and shield endothelial cells from hyperglycemia-induced damage effects that extend orexin’s regulatory reach beyond the central nervous system and pancreas.

In adipose tissue, orexin-A functions as a potent stimulator of glucose uptake and adipocyte maturation, mimicking key aspects of insulin signaling. Skrzypski et al. (2011) demonstrated in both 3T3-L1 murine adipocytes and primary rat adipocytes that orexin-A activates the PI3K pathway to promote GLUT4 translocation to the plasma membrane, thereby increasing glucose uptake in a dose-dependent manner. This signaling cascade extends downstream to activate peroxisome proliferator-activated receptor gamma (PPARγ), a master regulator of adipogenesis, which in turn upregulates CCAAT/enhancer-binding protein alpha (C/EBPα), driving lipid accumulation, triglyceride synthesis, and adiponectin secretion. Notably, orexin-A also suppresses lipolysis, further favoring energy storage. These findings position orexin as an insulin-like hormone in adipose tissue, capable of enhancing peripheral glucose clearance and improving systemic insulin sensitivity, particularly under conditions of positive energy balance.

In the vascular endothelium, orexin-A emerges as a defender against diabetic complications. Zhang et al. (2019) exposed human aortic endothelial cells to high-glucose conditions and found that orexin-A treatment significantly attenuates oxidative stress and inflammasome activation. Specifically, orexin-A activates SIRT1 (sirtuin 1), a NAD^+^-dependent deacetylase, which suppresses reactive oxygen species (ROS) generation via inhibition of NADPH oxidase 4 (NOX4) and thioredoxin-interacting protein (TxNIP). Downstream, SIRT1 blocks the NLRP3 inflammasome and reduces secretion of pro-inflammatory cytokines IL-1β and IL-18, while deacetylating NF-κB to prevent its nuclear translocation. These anti-inflammatory and antioxidant effects protect endothelial function, reduce vascular stiffness, and mitigate the progression of diabetic vasculopathy, suggesting orexin as a therapeutic shield in hyperglycemia-driven cardiovascular risk.

**FIGURE 5:**
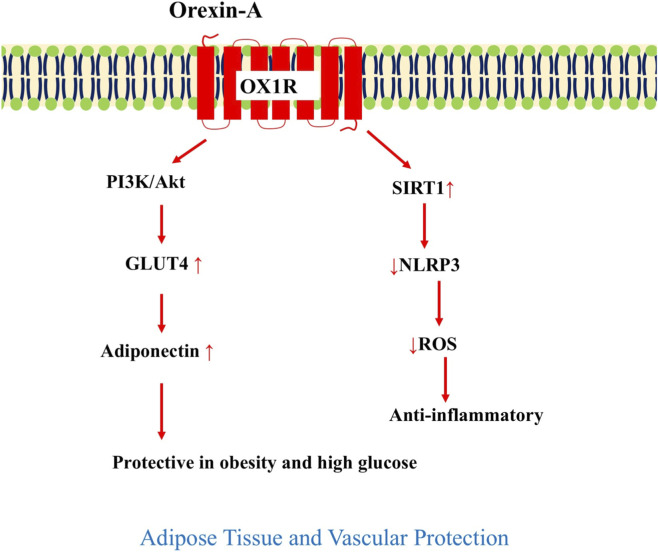
In adipocytes and endothelial cells, orexin-A signaling via OX1R promotes glucose uptake, adiponectin secretion, and anti-inflammatory effects under high-glucose conditions. OX1R, orexin receptor 1; PI3K, phosphoinositide 3-kinase; Akt, protein kinase B; GLUT4, glucose transporter type 4; PPARγ, peroxisome proliferator-activated receptor γ; C/EBPα, CCAAT/enhancer-binding protein α; SIRT1, sirtuin 1; NLRP3, NOD-, LRR- and pyrin domain-containing protein 3; ROS, reactive oxygen species.

In summary, orexin exerts insulin-like effects in adipose tissue through PI3K/GLUT4/PPARγ/C/EBPα signaling, promoting glucose uptake, lipid storage, and adiponectin release, while in vascular endothelium, it activates SIRT1 to suppress ROS, NLRP3, and NF-κB, conferring protection against diabetic endothelial dysfunction (Figure 5). Together, these actions enhance peripheral glucose utilization and reduce vascular inflammation, supporting orexin modulation as a strategy to combat insulin resistance and cardiovascular complications in metabolic syndrome.

### 3.5 Gut-orexin-glucose axis

A single but mechanistically rich study establishes the gut as a direct target of orexin signaling in the control of nutrient absorption, revealing a postprandial braking mechanism that limits glucose influx and prevents glycemic spikes. Ducroc et al. (2007) used Ussing chamber preparations of rat jejunum and *in vivo* oral glucose tolerance tests to demonstrate that both orexin-A and orexin-B potently inhibit intestinal glucose transport by 53%–59%. This suppression occurs through dual receptor-specific pathways: orexin-B acts directly on epithelial cells via OX1R to reduce sodium-glucose cotransporter 1 (SGLT-1) activity at the brush border, while orexin-A engages OX2R on enteric neurons and endocrine cells, triggering a neuronal-endocrine loop involving cholecystokinin (CCK) and tetrodotoxin (TTX)-sensitive neural transmission. The combined effect significantly lowers the short-circuit current (Isc) associated with electrogenic glucose absorption and attenuates the postprandial blood glucose rise *in vivo*. This inhibition is rapid, reversible, and independent of insulin, indicating a direct enteroregulatory role for orexin in delaying nutrient entry during feeding, complementary to its central orexigenic drive.

In summary, orexin delays the postprandial glucose surge by inhibiting intestinal SGLT-1-mediated absorption through OX1R (direct epithelial) and OX2R (neuronal/CCK-dependent) pathways. This gut-level glucose gating represents a previously underappreciated peripheral arm of orexin action, offering a novel therapeutic avenue, such as OX2R agonists, to slow carbohydrate absorption and improve glycemic control in diabetes, akin to α-glucosidase inhibitors but via a neuropeptide mechanism.

### 3.6 Multi-system or whole-body effects

Three studies bridged central and peripheral findings to reveal orexin’s systemic influence on glucose homeostasis, demonstrating that orexin deficiency precipitates global insulin resistance while orexin administration or restoration improves glycemic control across fasting, diabetic, and knockout models. Notably, this category includes the only human evidence in the review, providing critical translational insight into orexin’s clinical relevance.

In a landmark clinical investigation, Zarifkar et al. (2017) enrolled medication-naïve patients with newly diagnosed type 2 diabetes mellitus and measured serum orexin-A levels alongside insulin resistance indices. They observed a strong inverse correlation between circulating orexin-A and HOMA-IR, with diabetic patients exhibiting significantly lower orexin than healthy controls. Following 3 months of standard therapy with either metformin or pioglitazone, orexin-A levels rose by 26% and 14%, respectively, in parallel with improved insulin sensitivity and reduced fasting glucose. This human data firmly establishes that hypo-orexinemia accompanies insulin resistance and that pharmacological restoration of glycemic control upregulates orexin, suggesting a bidirectional, self-reinforcing loop between orexin signaling and metabolic health.

At the preclinical level, Tsuneki et al. (2002) administered orexin-A and orexin-B intravenously or intracerebroventricularly to both normal and streptozotocin-induced diabetic mice under fasting conditions. Both peptides produced a rapid, dose-dependent reduction in blood glucose, with effects persisting in the diabetic state and occurring independently of insulin secretion. The glucose-lowering action was attributed to enhanced peripheral glucose utilization via neural and hormonal pathways, including sympathetic activation of muscle glucose uptake and possible direct effects on hepatic metabolism, demonstrating orexin’s hypoglycemic potency even in insulin-deficient models.

Rounding out the systemic perspective, Devère et al. (2025) performed a comprehensive metabolic phenotyping of prepro-orexin knockout mice. These animals displayed profound glucose intolerance, impaired insulin secretion and sensitivity, and elevated hepatic gluconeogenesis, evidenced by increased expression of PEPCK and G6Pase alongside reduced glucokinase (GCK) activity. The multi-organ dysfunction spanning the hypothalamus, liver, pancreas, and adipose underscored orexin’s role as a master regulator of metabolic integration.

In summary, orexin deficiency drives global insulin resistance through disrupted central-peripheral coupling, as shown in orexin knockout models and human type 2 diabetes, where low orexin predicts high HOMA-IR. Conversely, orexin administration lowers blood glucose in fasting and diabetic states via insulin-independent utilization, and glycemic improvement restores orexin levels. These findings confirm an inverse orexin–insulin resistance axis in humans and animals, positioning orexin as a diagnostic biomarker and therapeutic target for metabolic syndrome.

### 3.7 Sexual dimorphism in the orexin–glucose axis

Detailed reviews of the available literature on the role of sex steroid hormones on metabolic changes (Kampire et al., 2025) and the orexinergic-glucose pathway (Hakizimana and Alagbonsi, 2025) have been recently published by our team. Although the majority of the included studies were conducted exclusively in male rodents or did not report animal sex, emerging evidence highlights a pronounced sexual dimorphism in orexin-mediated glucose homeostasis, with males exhibiting greater metabolic vulnerability. In orexin knockout and orexin-neuron-ablated mice, male animals develop significantly more severe late-onset obesity, hepatic steatosis, glucose intolerance, and insulin resistance than age-matched females under both standard chow and high-fat diet conditions (Tsuneki et al., 2008). This male-biased phenotype is testosterone-dependent, being completely abolished by gonadectomy and restored by androgen replacement, whereas ovariectomy has minimal impact (Tsuneki et al., 2008). Consistent with these preclinical observations, clinical data reveal that the inverse correlation between circulating orexin-A and insulin resistance (HOMA-IR) is observed predominantly in men, whereas postmenopausal women exhibit a decline in plasma orexin-A that is partially reversed by hormone replacement therapy, accompanied by improved fasting glucose (Zarifkar et al., 2017). These findings indicate that estrogen amplifies protective orexin signaling in key metabolic tissues, conferring relative resistance to insulin resistance and β-cell failure in females. Accordingly, orexin-based therapeutics are likely to require sex-specific dosing strategies and stratified clinical trial designs.

### 3.8 Overall synthesis of findings

Across the 30 studies, orexin emerges as a central-peripheral integrator of glucose homeostasis, executing dual, context-dependent roles: mobilizing glucose during energy deficit (via hypoglycemia-activated arousal, feeding, and sympathetic EGP) and enhancing insulin sensitivity during excess (through β-cell support, hepatic rhythmicity, adipose glucose uptake, and vascular protection). This duality is mediated predominantly by OX1R-dominant signaling converging on key transcription factors FOXO1 (suppressed for survival and gluconeogenic restraint), PGC-1α (downregulated in hepatic antagonism), CREB (activated in insulin secretion), and c-Fos (upregulated in counter-regulatory activation).

Therapeutically, OX1R-selective agonists hold promise for β-cell rescue and hypoglycemia countermeasures, while timed DORAs, such as suvorexant, offer a novel approach to nocturnal hepatic insulin resistance in type 2 diabetes. The human evidence of hypo-orexinemia in insulin resistance and its reversal with treatment further validates orexin as a clinically actionable target. Future research should prioritize longitudinal human studies, receptor-specific pharmacodynamics, and sex-specific effects to fully translate these mechanistic insights into precision metabolic therapies.

## 4 Discussion

### 4.1 Summary of findings

This systematic review of 30 studies establishes the orexinergic pathway as a central-peripheral integrator of glucose homeostasis with context-dependent duality: mobilizing glucose via arousal, feeding, and sympathetic endogenous glucose production (EGP) in hypoglycemia, while enhancing insulin sensitivity, β-cell function, and peripheral utilization in hyperglycemia. Central LH orexin neurons exhibit non-metabolic GI sensing (K_2_P/SGLT; ATP/AMP-independent), activated by NTS afferents during low glucose to upregulate c-Fos/CREB and drive VTA reward and ANS outputs. Peripherally, OX1R-dominant signaling converges on key transcription factors (TFs): reduced FOXO1 (β-cell survival, hepatic oxidation), CREB (glucose-stimulated insulin secretion [GSIS]), PGC-1α (gluconeogenesis restraint), C/EBPα (adipose uptake), and NF-κB (vascular protection). Systemic deficiency induces insulin resistance (human inverse orexin-HOMA-IR link), with male-biased severity in knockouts. These glucose effects are amplified by non-glucose outcomes: wakefulness, motivated feeding, anti-inflammation, and neuroprotection, positioning orexin as a multi-functional therapeutic target amid the 589 million diabetes burden (International Diabetes Federation, 2025).

### 4.2 Mechanistic integration: glucose sensing and tissue-specific signaling

Central to orexin’s glucose regulation is its extracellular, non-metabolic sensing in LH neurons, where physiological glucose (1–5 mM) hyperpolarizes cells via tandem-pore K^+^ (K_2_P) channels, independent of ATP, AMP, or lactate surrogates (Burdakov et al., 2006; González et al., 2008). This GI dominance, confirmed by 2-deoxyglucose mimicry and glucokinase inhibitor insensitivity, resolves apparent heterogeneity: rare glucose-excited (GE) responses occur in adjacent circuits (e.g., arcuate NPY/AgRP neurons) but not orexin cells, enabling predictive suppression of arousal during energy abundance (González et al., 2009b). Hypoglycemia, conversely, triggers rapid activation via NTS vagal afferents, upregulating c-Fos (33%) and prepro-orexin mRNA within hours (Cai et al., 2001; Moriguchi et al., 1999), driving wakefulness (48% increase) and sympathetic EGP (Tkacs et al., 2007; Yi et al., 2009). This aligns with evolutionary models tracing orexin from arousal peptides to metabolic alarms Inutsuka and Yamanaka, 2013), contrasting with slower, ATP-dependent sensing in pancreatic β-cells (Figure 3).

In comparison to prior reviews, Tsuneki et al. (2010) emphasized orexin’s bidirectional effects (glucose-elevating or -lowering depending on context), a duality our synthesis reinforces through 15 central studies, extending their focus on hypothalamic insulin/leptin interactions to include VTA reward circuits (Teegala et al., 2023). Similarly, Tsuneki et al. (2012) highlighted orexin’s role in energy homeostasis, but our analysis advances this by quantifying TF convergence, for instance, c-Fos in 40% of hypoglycemia models, addressing gaps in transcriptional detail. Adeghate et al. (2020) reviewed orexin’s modulation of insulin and glucagon in the diabetic pancreas. Yet, our inclusion of 6 β-cell studies reveals stronger OX1R/PI3K/Akt/FOXO1 suppression for proliferation, which is absent in their narrative that overlooked recent ERK/Elk-1 data (Skrzypski et al., 2016). Rani et al. (2018). Focused on central-peripheral orexin roles, but our 30-study scope surpasses their 20 citations, incorporating gut SGLT-1 inhibition (Ducroc et al., 2007) as a novel postprandial brake, contrasting their emphasis on pancreatic co-localization.

**FIGURE 6:**
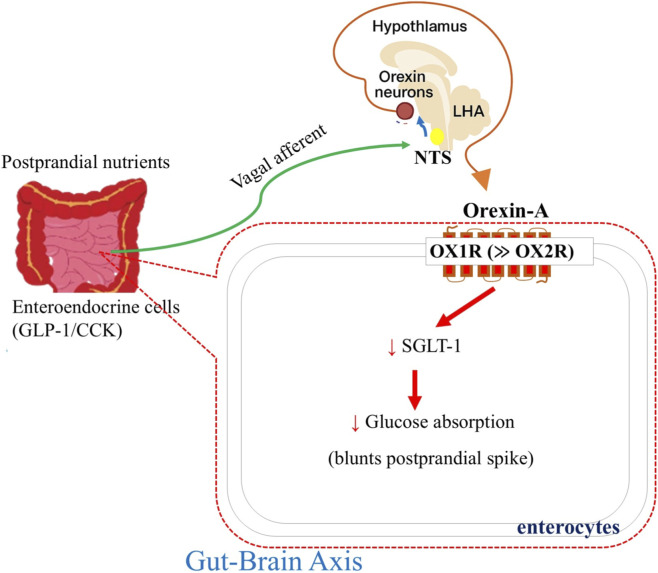
Nutrients stimulate enteroendocrine release of GLP-1 and CCK, which activate orexin neurons via vagal-NTS pathways. In turn, orexin acts on epithelial OX1R/OX2R to reduce SGLT1-mediated glucose absorption, blunting postprandial glycemic excursions. GLP-1, glucagon-like peptide-1; CCK, cholecystokinin; NTS, nucleus of the solitary tract; OX1R/OX2R, orexin receptors 1 and 2; SGLT1, sodium–glucose cotransporter 1.

Peripherally, orexin converges on TFs to execute tissue-specific effects (Figures 4–6). In the pancreas, OX1R/PI3K/Akt suppresses FOXO1 to enhance β-cell survival, while ERK/Elk-1/c-Fos drives proliferation, and cAMP/EPAC2/CREB amplifies GSIS (Chen et al., 2013; Park et al., 2015; Skrzypski et al., 2016). This mirrors GLP-1’s incretin action but adds central feedback, with low-glucose orexin-A favoring glucagon (Ouedraogo et al., 2003). Compared to Rani et al. (2018), who noted orexin’s pancreatic expression, our synthesis highlights its glucagon bias in fasting, extending their hypothesis on orexin as a “hormone-like” regulator. In the liver, PI3K/Akt/mTOR represses FOXO1/PGC-1α to shift flux toward oxidation, while ANS rhythmicity prevents insulin resistance (Tsuneki et al., 2015; Liu et al., 2016), a circadian mechanism absent in metformin, as critiqued in the work of Kalsbeek et al. (2010) for hypothalamic clock dysregulation. Adipose tissue uses PI3K/GLUT4/PPARγ/C/EBPα activation for uptake and adiponectin secretion (Skrzypski et al., 2011), and vascular endothelium benefits from SIRT1-mediated NLRP3/NF-κB inhibition under high glucose (Zhang et al., 2019). The gut axis, via OX1R/OX2R and CCK, reduces SGLT-1 absorption by 53%–59% (Ducroc et al., 2007), complementing α-glucosidase inhibitors. Systemic deficiency induces insulin resistance via disrupted Akt/GSK3β, with human type 2 diabetes (T2D) showing inverse orexin-HOMA-IR correlation (Zarifkar et al., 2017; Devère et al., 2025).

### 4.3 Non-glucose-related outcomes of orexin signaling

In addition to its metabolic effects, orexin signaling regulates several non-glucose-related outcomes, including sleep-wake cycles, arousal, reward processing, emotion for instance, anxiety, fear), and cognition, like attention and memory. For instance, orexin promotes wakefulness and stabilizes sleep transitions via projections to arousal centers like the locus coeruleus and dorsal raphe, with deficiencies causing narcolepsy-like symptoms such as cataplexy and fragmented sleep (Sakurai, 2007; Scammell et al., 2017). In reward, orexin modulates dopaminergic activity in the VTA through OX1R, contributing to addiction behaviors (Harris et al., 2005; Aston-Jones et al., 2010). Emotionally, it influences fear and mood via limbic connections, particularly through amygdala and prefrontal cortex interactions (Lungwitz et al., 2012; Flores et al., 2015), while cognitively, it supports executive function tied to arousal states, such as attention and working memory (Lambe et al., 2005; Yang et al., 2013).

Comparing mechanisms, glucose-related outcomes (e.g., insulin secretion via PI3K/Akt/mTOR in β-cells, GLUT4 translocation via AMPK/PI3K in muscle) share core GPCR pathways with non-glucose roles, such as Gq/PLC/IP368-mediated Ca^2+^ elevation and mTOR activation for cellular processes like autophagy and protein synthesis (Tsuneki et al., 2010; Leonard and Kukkonen, 2014). However, non-glucose mechanisms emphasize Gi/o coupling for cAMP inhibition (e.g., in OX2R for sleep regulation) and β-arrestin-biased signaling for sustained ERK in reward/emotion (Milasta et al., 2005; Yamanaka et al., 2010), whereas glucose homeostasis often involves Gαs/cAMP stimulation (e.g., for steroidogenesis linking to metabolic stress) and direct metabolic effectors like PEPCK/G6Pase (Tsuneki et al., 2002; Yi et al., 2009). Overlaps occur in mTORC1’s role in integrating energy states with arousal (e.g., via Ca^2+^-dependent v-ATPase) (Tsuneki et al., 2010), but non-glucose actions favor central neuronal excitability over peripheral anabolic responses, with heteromerization (e.g., with CB1 or CRF receptors) enabling context-specific modulation absent in pure glucose pathways (Navarro et al., 2015; Olney et al., 2017). These non-glucose roles enhance the understanding of orexin’s integrative function, as arousal and circadian alignment (e.g., via suprachiasmatic nucleus [SCN] inputs) directly influence metabolic outcomes like glucose tolerance, while reward and emotional regulation may indirectly affect feeding behaviors, linking to obesity and diabetes risk (Harris et al., 2005; Tsuneki et al., 2016). Compared to Adeghate (2020), which linked orexin to obesity via energy expenditure, our review integrates these with glucose duality, showing how arousal-feeding loops (Tkacs et al., 2007) amplify glycemic defense a connection underexplored in their pancreatic focus.

### 4.4 Sexual dimorphism and translational implications

The orexinergic regulation of glucose homeostasis displays marked sexual dimorphism, with males showing greater susceptibility to the deleterious metabolic consequences of orexin deficiency than females. This dimorphism arises from opposing actions of gonadal steroids: testosterone enhances orexin neuron excitability and promotes insulin resistance upon orexin loss, whereas 17β-estradiol upregulates OX1R expression and potentiates insulin-sensitizing and β-cell-protective effects of orexin-A (Tsuneki et al., 2008; Hakizimana and Alagbonsi, 2025). These preclinical observations align with human data and mirror the sex-specific efficacy and safety profiles already documented for GLP-1 receptor agonists and SGLT2 inhibitors. Consequently, orexin receptor modulators, whether OX1R/OX2R agonists for hypoglycemia unawareness and type 1 diabetes or dual antagonists for nocturnal hyperglycemia in type 2 diabetes, will likely require sex-specific dosing, timing, or formulation. Future clinical development programs must therefore incorporate sex stratification from phase 2 onward and consider menopausal/androgen status to maximize therapeutic benefit and avoid efficacy biases currently observed in metabolic pharmacotherapy.

### 4.5 Comparison with other studies

Unlike previous reviews that broadly addressed orexin’s role in energy expenditure, arousal, or behavioral regulation, this systematic review is unique in demonstrating an in-depth, tissue-specific modulation of glucose homeostasis by orexin. For example, the review by Tsuneki et al. (2012) mainly examined the physiological functions of orexin without detailing the subsequent intracellular signaling cascades in peripheral tissues. Likewise, Tsuneki et al. (2010) and Sakurai (2005) presented the regulation of hunger and central orexin activity, but little about its direct metabolic consequences. Rani et al. (2018) discussed more general neuropeptide functions without providing detailed information on glucose metabolism. Messina (2014) and Xiao et al. (2021) tersely touched on the metabolic role of orexin without tissue specificity or experimental focus.

In contrast, this analysis is the first to track the unique roles of orexin in the pancreas, liver, muscle, adipose tissue, and GI tract by organizing and compiling experimental papers (n = 30) from a variety of models, including cell lines, animal studies, and limited human data. Unlike Milbank and López (2019) and Mogavero et al. (2023), our research provides comprehensive cellular and molecular frameworks (such as PI3K/Akt/mTOR pathways, OX1R/OX2R signaling, and GLUT4 trafficking), providing a mechanistically grounded basis for upcoming clinical translation. This review presents comprehensive cellular and molecular evidence of orexin’s metabolic effects on glucose homeostasis, contrasting with previous reviews that have focused on behavioral and neuroendocrine consequences (Sakurai, 2014; Kukkonen, 2019). The findings expand on the results of a previous review by Messina (2014), who highlighted the role of neuropeptides in metabolic control, but only included orexin without providing details. This review uniquely maps orexin’s tissue-specific roles mechanistically, contrasting prior broad overviews (Sakurai, 2005; Tsuneki et al., 2010) by detailing pathways like OX1R/PI3K in pancreas versus SNS/β2-AR in muscle. Furthermore, by integrating non-glucose outcomes (sleep, reward), this review extends prior work by showing how orexin’s broader roles (circadian alignment, feeding behavior) complement its metabolic functions, offering a holistic view for therapeutic targeting (Harris et al., 2005; Sakurai, 2007).

### 4.6 Clinical implications

Both clinical practice and public health policy are significantly impacted by the evidence presented in this review. Orexin is a promising therapeutic target for the management of insulin resistance and diabetes because of its function in regulating peripheral glucose uptake throughout tissues, hepatic glucose synthesis, and insulin secretion. To improve β-cell function and promote insulin-independent glucose absorption, specifically in people with defective insulin signaling, orexin receptor agonists may be developed (Chen et al., 2013; Liu et al., 2016; Skrzypski et al., 2016).

On the other hand, preclinical models have shown that the strategic use of dual orexin receptor antagonists (DORAs), such as suvorexant, may improve metabolic circadian alignment and reduce nocturnal hyperglycemia (Tsuneki et al., 2016). Precision medicine approaches to diabetes treatment may be possible if orexin regulation is tailored to particular stages of the circadian cycle or metabolic state. The results encourage more funding for translational studies examining the metabolic processes of neuropeptides from a policy standpoint. Because orexin is involved in many different systems, therapeutic targeting may have repercussions on cardiovascular health, food habits, sleep quality, and glucose homeostasis (Sakurai, 2014). The significance of integrated care models and financing for cross-functional research projects is highlighted by this cross-disciplinary relevance.

A new treatment approach for diabetes and metabolic syndrome may be provided by modifying orexinergic transmission, given the consistent metabolic effects of orexin-A seen in preclinical research. Orexin agonists, for instance, might improve insulin sensitivity or β-cell function, whereas suvorexant and other circadian-timed antagonists might maximize glycemic cycles. Clinical trials should be conducted to investigate these findings further. Though orexin-B regulates insulin, glucose uptake, and metabolic signaling, its metabolic role is less potent and underrepresented in current studies. Expanding therapeutic implications, DORAs (e.g., daridorexant, lemborexant) show potential beyond insomnia for metabolic disorders, improving glucose tolerance in obesity models by blocking OX2R-driven overeating and SNS hyperactivity. Selective OX1R antagonists target reward/metabolic imbalance in addiction-linked obesity, while agonists (e.g., orexin-A mimetics) could treat narcolepsy-associated IR by enhancing muscle uptake. Differential roles (OX1R for feeding/motivation, OX2R for arousal/thermogenesis) enable precision: OX1R agonists for hypoglycemia, DORAs for chrono-disrupted T2DM, and positioning orexin modulation as versatile for personalized metabolic therapy (Tsuneki et al., 2016; Kaczmarek et al., 2017).

### 4.7 Limitations and future directions

Limitations include preclinical dominance (29/30 studies), muscle underrepresentation (despite sympathetic GLUT4 effects; Shiuchi et al., 2009), and sparse human data. Sensing heterogeneity requires single-cell resolution and non-glucose outcomes like memory and inflammation. Need metabolic integration. Compared to Rani et al. (2018), our broader scope addresses peripheral gaps but shares their call for longitudinal human trials. Sex-stratified randomized controlled trials (RCTs) in LMICs, OX1R agonists for type 1 diabetes (T1D), and artificial intelligence-guided circadian orexin monitoring to address the $1.1 trillion annual burden (IDF, 2025) are recommended as future priorities.

## 5 Conclusion

In conclusion, orexin’s glucose regulation amplified by its roles in arousal, feeding, and inflammation redefines it as a metabolic conductor and crucial neuroendocrine regulator of glucose homeostasis, coordinating adaptive responses to energy demands through central glucose sensing and peripheral metabolic actions. Its therapeutic modulation could shift diabetes care from peripheral mitigation to central-peripheral harmony, making it a prospective target for metabolic treatments, especially in insulin resistance and diabetes, because of its capacity to react dynamically to internal signals. Translational studies are urgently needed to harness orexin pathways for therapeutic use in metabolic disease management and achieve human validation.

## Data Availability

The original contributions presented in the study are included in the article/Supplementary Material, further inquiries can be directed to the corresponding author.

## References

[B1] Adeghate E. Lotfy M. D'Souza C. Alseiari S. M. Alsaadi A. A. Qahtan S. A. (2020). Hypocretin/Orexin modulates body weight and the metabolism of glucose and insulin. Diabetes/Metabolism Res. Rev. 36 (3), e3229. 10.1002/dmrr.3229 31655012

[B2] Alonge K. M. D’Alessio D. A. Schwartz M. W. (2021). Brain control of blood glucose levels: implications for the pathogenesis of type 2 diabetes. Diabetologia 64 (1), 5–14. 10.1007/s00125-020-05293-3 33043401 PMC7718404

[B3] Aston-Jones G. Smith R. J. Sartor G. C. Moorman D. E. Massi L. Tahsili-Fahadan P. (2010). Lateral hypothalamic orexin/hypocretin neurons: a role in reward-seeking and addiction. Brain Res. 1314, 74–90. 10.1016/j.brainres.2009.09.106 19815001 PMC2819557

[B76] Bae C. Song J. (2017). The role of glucagon-like peptide 1 (GLP1) in type 3 diabetes: GLP-1 controls insulin resistance, neuroinflammation and neurogenesis in the brain. Int. J. Mol. Sci. 18 (11), 2493. 10.3390/ijms18112493 29165354 PMC5713459

[B4] Barson J. R. Leibowitz S. F. (2017). “Orexin/hypocretin system: role in food and drug overconsumption,” in International review of neurobiology (Elsevier), 199–237. 10.1016/bs.irn.2017.06.006 PMC5820772 29056152

[B5] Burdakov D. Jensen L. T. Alexopoulos H. Williams R. H. Fearon I. M. O'Kelly I. (2006). Tandem-pore K+ channels mediate inhibition of orexin neurons by glucose. Neuron 50 (5), 711–722. 10.1016/j.neuron.2006.04.032 16731510

[B6] Cai X. J. Evans M. L. Lister C. A. Leslie R. A. Arch J. R. Wilson S. (2001). Hypoglycemia activates orexin neurons and selectively increases hypothalamic orexin-B levels: responses inhibited by feeding and possibly mediated by the nucleus of the solitary tract. Diabetes 50 (1), 105–112. 10.2337/diabetes.50.1.105 11147774

[B7] Chen L. Zhao Y. Zheng D. Ju S. Shen Y. Guo L. (2013). Orexin A affects INS-1 rat insulinoma cell proliferation *via* orexin receptor 1 and the AKT signaling pathway. Int. J. Endocrinol. 2013, 1–7. 10.1155/2013/854623 24382962 PMC3871501

[B8] Choi J. H. Kim M.-S. (2022). Homeostatic regulation of glucose metabolism by the central nervous system. Endocrinol. Metabolism 37 (1), 9–25. 10.3803/EnM.2021.1364 35255598 PMC8901968

[B9] Coppari R. (2015). Hypothalamic neurones governing glucose homeostasis. J. Neuroendocrinol. 27 (6), 399–405. 10.1111/jne.12276 25778859

[B10] Devère M. Takhlidjt S. Godefroy D. do Rego J. L. do Rego J. C. Bénani A. (2025). Glucose and energy metabolism are impaired in mice deficient for orexins. J. Endocrinol. 265 (2), e240329. 10.1530/JOE-24-0329 40066929

[B11] Diepenbroek C. Serlie M. J. Fliers E. Kalsbeek A. la Fleur S. E. (2013). Brain areas and pathways in the regulation of glucose metabolism. BioFactors 39 (5), 505–513. 10.1002/biof.1123 23913677

[B12] Ducroc R. Voisin T. El Firar A. Laburthe M. (2007). Orexins control intestinal glucose transport by distinct neuronal, endocrine, and direct epithelial pathways. Diabetes 56 (10), 2494–2500. 10.2337/db07-0614 17626888 PMC2214858

[B13] Flores Á. Saravia R. Maldonado R. Berrendero F. (2015). Orexins and fear: implications for the treatment of anxiety disorders. Trends Neurosci. 38 (9), 550–559. 10.1016/j.tins.2015.06.005 26216377

[B14] González J. A. Jensen L. T. Fugger L. Burdakov D. (2008). Metabolism-independent sugar sensing in central orexin neurons. Diabetes 57 (10), 2569–2576. 10.2337/db08-0548 18591392 PMC2551664

[B15] Gonzàlez J. A. Reimann F. Burdakov D. (2009a). Dissociation between sensing and metabolism of glucose in sugar sensing neurones. J. Physiology 587 (1), 41–48. 10.1113/jphysiol.2008.163410 18981030 PMC2670021

[B16] González J. A. Jensen L. T. Doyle S. E. Miranda-Anaya M. Menaker M. Fugger L. (2009b). Deletion of TASK1 and TASK3 channels disrupts intrinsic excitability but does not abolish glucose or pH responses of orexin/hypocretin neurons. Eur. J. Neurosci. 30 (1), 57–64. 10.1111/j.1460-9568.2009.06789.x 19508695 PMC3410734

[B17] Griffond B. Risold P. Y. Jacquemard C. Colard C. Fellmann D. (1999). Insulin-induced hypoglycemia increases preprohypocretin (orexin) mRNA in the rat lateral hypothalamic area. Neurosci. Lett. 262 (2), 77–80. 10.1016/S0304-3940(98)00976-8 10203235

[B18] Hakizimana J. C. Alagbonsi A. I. (2025). Modulation of lactose synthesis and orexinergic‐glucose pathway by sex steroid hormones. Physiol. Rep. 13 (22), e70661. 10.14814/phy2.70661 41243426 PMC12620397

[B19] Harris G. C. Wimmer M. Aston-Jones G. (2005). A role for lateral hypothalamic orexin neurons in reward seeking. Nature 437 (7058), 556–559. 10.1038/nature04071 16100511

[B20] Hooijmans C. R. Rovers M. M. de Vries R. B. M. Leenaars M. Ritskes-Hoitinga M. Langendam M. W. (2014). SYRCLE’s risk of bias tool for animal studies. BMC Med. Res. Methodol. 14 (1), 43. 10.1186/1471-2288-14-43 24667063 PMC4230647

[B21] Inoki K. Kim J. Guan K.-L. (2012). AMPK and mTOR in cellular energy homeostasis and drug targets. Annu. Rev. Pharmacol. Toxicol. 52 (1), 381–400. 10.1146/annurev-pharmtox-010611-134537 22017684

[B77] Inutsuka A. Yamanaka A. (2013). The physiological role of orexin/hypocretin neurons in the regulation of sleep/wakefulness and neuroendocrine functions. Front. Endocrinol. 4. 10.3389/fendo.2013.00018 23508038 PMC3589707

[B22] International Diabetes Federation (2025). IDF diabetes atlas 2025. Diabetes Atlas. Available online at: https://diabetesatlas.org/resources/idf-diabetes-atlas-2025/(Accessed: June 20, 2025).

[B23] Jöhren O. Neidert S. J. Kummer M. Dendorfer A. Dominiak P. (2001). Prepro-orexin and orexin receptor mRNAs are differentially expressed in peripheral tissues of Male and female rats. Endocrinology 142 (8), 3324–3331. 10.1210/endo.142.8.8299 11459774

[B24] Kaczmarek P. Skrzypski M. Pruszynska-Oszmalek E. Sassek M. Kolodziejski P. A. Billert M. (2017). Chronic orexin-A (hypocretin-1) treatment of type 2 diabetic rats improves glucose control and beta-cell functions. J. Physiology Pharmacol. 68 (5), 669–681. 29375041

[B25] Kalsbeek A. Yi C. X. La Fleur S. E. Fliers E. (2010). The hypothalamic clock and its control of glucose homeostasis. Trends Endocrinol. and Metabolism 21 (7), 402–410. 10.1016/j.tem.2010.02.005 20303779

[B26] Kampire M. G. Hakizimana J. C. Mucumbitsi J. Alagbonsi A. I. (2025). Pathophysiological consequences associated with hormonal contraceptives use in Sub-Saharan Africa: a scoping review. Open Access J. Contraceptives 16, 171–187. 10.2147/OAJC.S563680 41262159 PMC12625715

[B27] Kukkonen J. P. (2019). “Pharmacology of orexin/hypocretin receptors,” in The orexin/hypocretin system (Elsevier), 31–68. 10.1016/B978-0-12-813751-2.00002-4

[B28] Lambe E. K. Olausson P. Horst N. K. Taylor J. R. Aghajanian G. K. (2005). Hypocretin and nicotine excite the same thalamocortical synapses in prefrontal cortex: correlation with improved attention in rat. J. Neurosci. 25 (21), 5225–5229. 10.1523/JNEUROSCI.0719-05.2005 15917462 PMC6724823

[B29] Leonard C. S. Kukkonen J. P. (2014). Orexin/Hypocretin receptor signalling: a functional perspective. Br. J. Pharmacol. 171 (2), 294–313. 10.1111/bph.12296 23848055 PMC3904253

[B30] Liu X. H. Morris R. Spiller D. White M. Williams G. (2001). Orexin a preferentially excites glucose-sensitive neurons in the lateral hypothalamus of the rat *in vitro* . Diabetes 50 (11), 2431–2437. 10.2337/diabetes.50.11.2431 11679418

[B31] Liu Y. Zhao Y. Guo L. (2016). Effects of orexin A on glucose metabolism in human hepatocellular carcinoma *in vitro via* PI3K/Akt/mTOR-dependent and -independent mechanism. Mol. Cell. Endocrinol. 420, 208–216. 10.1016/j.mce.2015.11.002 26549689

[B32] Lungwitz E. A. Molosh A. Johnson P. L. Harvey B. P. Dirks R. C. Dietrich A. (2012). Orexin-A induces anxiety-like behavior through interactions with glutamatergic receptors in the bed nucleus of the stria terminalis of rats. Physiology and Behav. 107 (5), 726–732. 10.1016/j.physbeh.2012.05.019 22652097 PMC3482273

[B33] Messina G. (2014). Orexin A controls glucose metabolism. J. Diabetes and Metabolism 05 (07). 10.4172/2155-6156.1000398

[B34] Milasta S. Evans N. A. Ormiston L. Wilson S. Lefkowitz R. J. Milligan G. (2005). The sustainability of interactions between the orexin-1 receptor and β-arrestin-2 is defined by a single C-terminal cluster of hydroxy amino acids and modulates the kinetics of ERK MAPK regulation. Biochem. J. 387 (3), 573–584. 10.1042/BJ20041745 15683363 PMC1134986

[B35] Milbank E. López M. (2019). Orexins/hypocretins: key regulators of energy homeostasis. Front. Endocrinol. 10, 830. 10.3389/fendo.2019.00830 31920958 PMC6918865

[B36] Minozzi S. Dwan K. Borrelli F. Filippini G. (2022). Reliability of the revised cochrane risk-of-bias tool for randomised trials (RoB2) improved with the use of implementation instruction. J. Clin. Epidemiol. 141, 99–105. 10.1016/j.jclinepi.2021.09.021 34537386

[B37] Mogavero M. P. Godos J. Grosso G. Caraci F. Ferri R. (2023). Rethinking the role of orexin in the regulation of REM sleep and appetite. Nutrients 15 (17), 3679. 10.3390/nu15173679 37686711 PMC10489991

[B38] Moriguchi T. Sakurai T. Nambu T. Yanagisawa M. Goto K. (1999). Neurons containing orexin in the lateral hypothalamic area of the adult rat brain are activated by insulin-induced acute hypoglycemia. Neurosci. Lett. 264 (1–3), 101–104. 10.1016/s0304-3940(99)00177-9 10320024

[B39] Navarro G. Quiroz C. Moreno-Delgado D. Sierakowiak A. McDowell K. Moreno E. (2015). Orexin–corticotropin-releasing factor receptor heteromers in the ventral tegmental area as targets for cocaine. J. Neurosci. 35 (17), 6639–6653. 10.1523/JNEUROSCI.4364-14.2015 25926444 PMC4412889

[B40] Nowak K. W. Maćkowiak P. Switońska M. M. Fabiś M. Malendowicz L. K. (1999). Acute orexin effects on insulin secretion in the rat: *in vivo* and *in vitro* studies. Life Sci. 66 (5), 449–454. 10.1016/S0024-3205(99)00611-6 10670833

[B41] Nowak K. Strowski M. Z. Switonska M. M. Kaczmarek P. Singh V. Fabis M. (2005). Evidence that orexins A and B stimulate insulin secretion from rat pancreatic islets *via* both receptor subtypes. Int. J. Mol. Med. 15, 969–972. 10.3892/ijmm.15.6.969 15870901

[B42] Olney J. J. Navarro M. Thiele T. E. (2017). The role of orexin signaling in the ventral tegmental area and central amygdala in modulating binge-like ethanol drinking behavior. Alcohol. Clin. Exp. Res. 41 (3), 551–561. 10.1111/acer.13336 28097729 PMC5332299

[B43] Otlivanchik O. Le Foll C. Levin B. E. (2015). Perifornical hypothalamic orexin and serotonin modulate the counterregulatory response to hypoglycemic and glucoprivic stimuli. Diabetes 64 (1), 226–235. 10.2337/db14-0671 25114294 PMC4274798

[B44] Ouedraogo R. Näslund E. Kirchgessner A. L. (2003). Glucose regulates the release of orexin-a from the endocrine pancreas. Diabetes 52 (1), 111–117. 10.2337/diabetes.52.1.111 12502500

[B45] Page M. J. McKenzie J. E. Bossuyt P. M. Boutron I. Hoffmann T. C. Mulrow C. D. (2021). The PRISMA 2020 statement: an updated guideline for reporting systematic reviews. BMJ 372, n71. 10.1136/bmj.n71 33782057 PMC8005924

[B46] Park J.-H. Shim H. M. Na A. Y. Bae J. H. Im S. S. Song D. K. (2015). Orexin A regulates plasma insulin and leptin levels in a time-dependent manner following a glucose load in mice. Diabetologia 58 (7), 1542–1550. 10.1007/s00125-015-3573-0 25813215

[B47] Rani M. Kumar R. Krishan P. (2018). Role of orexins in the central and peripheral regulation of glucose homeostasis: Evidences and mechanisms. Neuropeptides 68, 1–6. 10.1016/j.npep.2018.02.002 29472002

[B48] Sakurai T. (2005). Roles of orexin/hypocretin in regulation of sleep/wakefulness and energy homeostasis. Sleep. Med. Rev. 9 (4), 231–241. 10.1016/j.smrv.2004.07.007 15961331

[B49] Sakurai T. (2007). The neural circuit of orexin (hypocretin): maintaining sleep and wakefulness. Nat. Rev. Neurosci. 8 (3), 171–181. 10.1038/nrn2092 17299454

[B50] Sakurai T. (2014). The role of orexin in motivated behaviours. Nat. Rev. Neurosci. 15 (11), 719–731. 10.1038/nrn3837 25301357

[B51] Sakurai T. Amemiya A. Ishii M. Matsuzaki I. Chemelli R. M. Tanaka H. (1998). Orexins and orexin receptors: a family of hypothalamic neuropeptides and G protein-coupled receptors that regulate feeding behavior. Cell 92 (4), 573–585. 10.1016/S0092-8674(00)80949-6 9491897

[B52] Scammell T. E. Arrigoni E. Lipton J. O. (2017). Neural circuitry of wakefulness and sleep. Neuron 93 (4), 747–765. 10.1016/j.neuron.2017.01.014 28231463 PMC5325713

[B53] Scheen A. J. (2010). Central nervous system: a conductor orchestrating metabolic regulations harmed by both hyperglycaemia and hypoglycaemia. Diabetes and Metabolism 36, S31–S38. 10.1016/S1262-3636(10)70464-X 21211733

[B54] Shiuchi T. Haque M. S. Okamoto S. Inoue T. Kageyama H. Lee S. (2009). Hypothalamic orexin stimulates feeding-associated glucose utilization in skeletal muscle *via* sympathetic nervous system. Cell Metab. 10 (6), 466–480. 10.1016/j.cmet.2009.09.013 19945404

[B55] Skrzypski M. T Le T. Kaczmarek P. Pruszynska-Oszmalek E. Pietrzak P. Szczepankiewicz D. (2011). Orexin A stimulates glucose uptake, lipid accumulation and adiponectin secretion from 3T3-L1 adipocytes and isolated primary rat adipocytes. Diabetologia 54 (7), 1841–1852. 10.1007/s00125-011-2152-2 21505958

[B56] Skrzypski M. Khajavi N. Mergler S. Billert M. Szczepankiewicz D. Wojciechowicz T. (2016). Orexin a modulates INS-1E cell proliferation and insulin secretion *via* extracellular signal-regulated kinase and transient receptor potential channels. J. Physiology Pharmacol. 67 (5), 643–652. 28011945

[B57] Teegala S. B. Sheng Z. Dalal M. S. Hirschberg P. R. Beck K. D. Routh V. H. (2020). Lateral hypothalamic orexin glucose-inhibited neurons May regulate reward-based feeding by modulating glutamate transmission in the ventral tegmental area. Brain Res. 1731, 145808. 10.1016/j.brainres.2018.05.025 29787770 PMC6525648

[B58] Teegala S. B. Sarkar P. Siegel D. M. Sheng Z. Hao L. Bello N. T. (2023). Lateral hypothalamus hypocretin/orexin glucose-inhibited neurons promote food seeking after calorie restriction. Mol. Metab. 76, 101788. 10.1016/j.molmet.2023.101788 37536499 PMC10448466

[B59] Tkacs N. C. Pan Y. Sawhney G. Mann G. L. Morrison A. R. (2007). Hypoglycemia activates arousal-related neurons and increases wake time in adult rats. Physiology and Behav. 91 (2–3), 240–249. 10.1016/j.physbeh.2007.03.003 17434543 PMC1934507

[B60] Tricco A. C. Lillie E. Zarin W. O'Brien K. K. Colquhoun H. Levac D. (2018). PRISMA extension for scoping reviews (PRISMA-ScR): checklist and explanation. Ann. Intern. Med. 169 (7), 467–473. 10.7326/M18-0850 30178033

[B61] Tsuneki H. Sugihara Y. Honda R. Wada T. Sasaoka T. Kimura I. (2002). Reduction of blood glucose level by orexins in fasting normal and streptozotocin-diabetic mice. Eur. J. Pharmacol. 448 (2–3), 245–252. 10.1016/S0014-2999(02)01936-2 12144948

[B62] Tsuneki H. Murata S. Anzawa Y. Soeda Y. Tokai E. Wada T. (2008). Age-related insulin resistance in hypothalamus and peripheral tissues of orexin knockout mice. Diabetologia 51 (4), 657–667. 10.1007/s00125-008-0929-8 18256806

[B63] Tsuneki H. Wada T. Sasaoka T. (2010). Role of orexin in the regulation of glucose homeostasis. Acta Physiol. 198 (3), 335–348. 10.1111/j.1748-1716.2009.02008.x 19489767

[B64] Tsuneki H. Wada T. Sasaoka T. (2012). Role of orexin in the central regulation of glucose and energy homeostasis. Endocr. J. 59 (5), 365–374. 10.1507/endocrj.EJ12-0030 22293586

[B65] Tsuneki H. Tokai E. Nakamura Y. Takahashi K. Fujita M. Asaoka T. (2015). Hypothalamic orexin prevents hepatic insulin resistance *via* daily bidirectional regulation of autonomic nervous system in mice. Diabetes 64 (2), 459–470. 10.2337/db14-0695 25249578

[B66] Tsuneki H. Kon K. Ito H. Yamazaki M. Takahara S. Toyooka N. (2016). Timed inhibition of orexin system by suvorexant improved sleep and glucose metabolism in type 2 diabetic Db/db mice. Endocrinology 157 (11), 4146–4157. 10.1210/en.2016-1404 27631554

[B67] Xiao X. Yeghiazaryan G. Hess S. Klemm P. Sieben A. Kleinridders A. (2021). Orexin receptors 1 and 2 in serotonergic neurons differentially regulate peripheral glucose metabolism in obesity. Nat. Commun. 12 (1), 5249. 10.1038/s41467-021-25380-2 34475397 PMC8413382

[B68] Xu T.-R. Ward R. J. Pediani J. D. Milligan G. (2011). The orexin OX1 receptor exists predominantly as a homodimer in the basal state: potential regulation of receptor organization by both agonist and antagonist ligands. Biochem. J. 439 (1), 171–183. 10.1042/BJ20110230 21770891

[B69] Yamanaka A. Muraki Y. Ichiki K. Tsujino N. Kilduff T. S. Goto K. (2006). Orexin neurons are directly and indirectly regulated by catecholamines in a complex manner. J. Neurophysiology 96 (1), 284–298. 10.1152/jn.01361.2005 16611835

[B70] Yamanaka A. Tabuchi S. Tsunematsu T. Fukazawa Y. Tominaga M. (2010). Orexin directly excites orexin neurons through orexin 2 receptor. J. Neurosci. 30 (38), 12642–12652. 10.1523/JNEUROSCI.2120-10.2010 20861370 PMC6633594

[B71] Yang L. Zou B. Xiong X. Pascual C. Xie J. Malik A. (2013). Hypocretin/orexin neurons contribute to hippocampus-dependent social memory and synaptic plasticity in mice. J. Neurosci. 33 (12), 5275–5284. 10.1523/JNEUROSCI.3200-12.2013 23516292 PMC3640412

[B72] Yang D. Xu L. Guo F. Sun X. Zhang D. Wang M. (2018). Orexin-A and endocannabinoid signaling regulate glucose-responsive arcuate nucleus neurons and feeding behavior in Obese rats. Neuropeptides 69, 26–38. 10.1016/j.npep.2018.04.001 29678290

[B73] Yi C.-X. Serlie M. J. Ackermans M. T. Foppen E. Buijs R. M. Sauerwein H. P. (2009). A major role for perifornical orexin neurons in the control of glucose metabolism in rats. Diabetes 58 (9), 1998–2005. 10.2337/db09-0385 19592616 PMC2731521

[B74] Zarifkar M. Noshad S. Shahriari M. Afarideh M. Khajeh E. Karimi Z. (2017). Inverse association of peripheral Orexin-A with insulin resistance in type 2 diabetes mellitus: A randomized clinical trial. Rev. Diabet. Stud. 14 (2–3), 301–310. 10.1900/RDS.2017.14.301 29145540 PMC6115012

[B75] Zhang C. Abdukerim M. Abilailieti M. Tang L. Ling Y. Pan S. (2019). The protective effects of orexin a against high glucose-induced activation of NLRP3 inflammasome in human vascular endothelial cells. Archives Biochem. Biophysics 672, 108052. 10.1016/j.abb.2019.07.017 31351069

